# Complete Remission After Palliative Radiation and Chemotherapy in Stage IV BRCA1-Positive Pancreatic Cancer: A Case of a Patient Who Was Cured 11 Years After Treatment

**DOI:** 10.7759/cureus.98008

**Published:** 2025-11-28

**Authors:** Simranjit Kaur, Chrystal Huynh, Victoria Pan, Khalid Hirmiz

**Affiliations:** 1 Oncology, Windsor Regional Hospital, Windsor, CAN

**Keywords:** brca gene, chemotherapy, complete remission, palliative radiation, pancreatic cancer, prognosis

## Abstract

Pancreatic cancer is associated with high mortality rates due to its markedly aggressive nature and typically late diagnosis. This is especially true for stage IV, in which the prognosis averages only a few months of median survival. We report a case that highlights the benefits of offering palliative treatment for terminal-stage pancreatic cancer to achieve unexpectedly prolonged survival and potential cure. A complete response was observed following low-dose palliative radiation and chemotherapy in a 54-year-old female patient who was BRCA1-positive and presented with stage IV pancreatic adenocarcinoma. There was no residual cancer or further metastasis seen on follow-up computed tomography (CT) imaging. The patient remained cancer-free and asymptomatic 11 years after the initial diagnosis.

## Introduction

Pancreatic cancer is the seventh leading cause of cancer deaths globally [1]. With a high mortality rate due to its highly aggressive nature and typically late diagnosis, it is projected that pancreatic cancer will be diagnosed in over 7,000 people and will be the cause of over 6,000 deaths in Canada in 2024 [2]. Key risk factors include smoking, age over 55 years old, male sex, obesity, diabetes, chronic pancreatitis, family history, work exposures, and genetic predispositions such as BRCA1/BRCA2 mutations, which are found in approximately 10%-20% of patients and may influence treatment outcomes [1,3].

Tumour markers such as serum carbohydrate antigen (CA 19-9) are commonly elevated in pancreatic cancer, aiding in the diagnosis, prognosis, and monitoring of the disease, though limitations in sensitivity (79%-81%) and specificity (82%-90%) exist [4].

The median survival of a patient with stage IV pancreatic cancer is two to six months [4], with only 2% of patients surviving past five years in Canada [5]. Surgical resection is currently recognized as the only definitive cure; however, merely 20% of pancreatic cancers are surgical candidates at the time of diagnosis due to the extent of distant metastasis and local spread [1]. 

We report a case of stage IV pancreatic adenocarcinoma that was treated with low-dose palliative radiation and chemotherapy. After 11 years, there was no sign of any cancer on computed tomography (CT) imaging and no elevations in CA 19-9, which led the team to believe that this patient had achieved complete remission (CR).

## Case presentation

**Figure 1:**
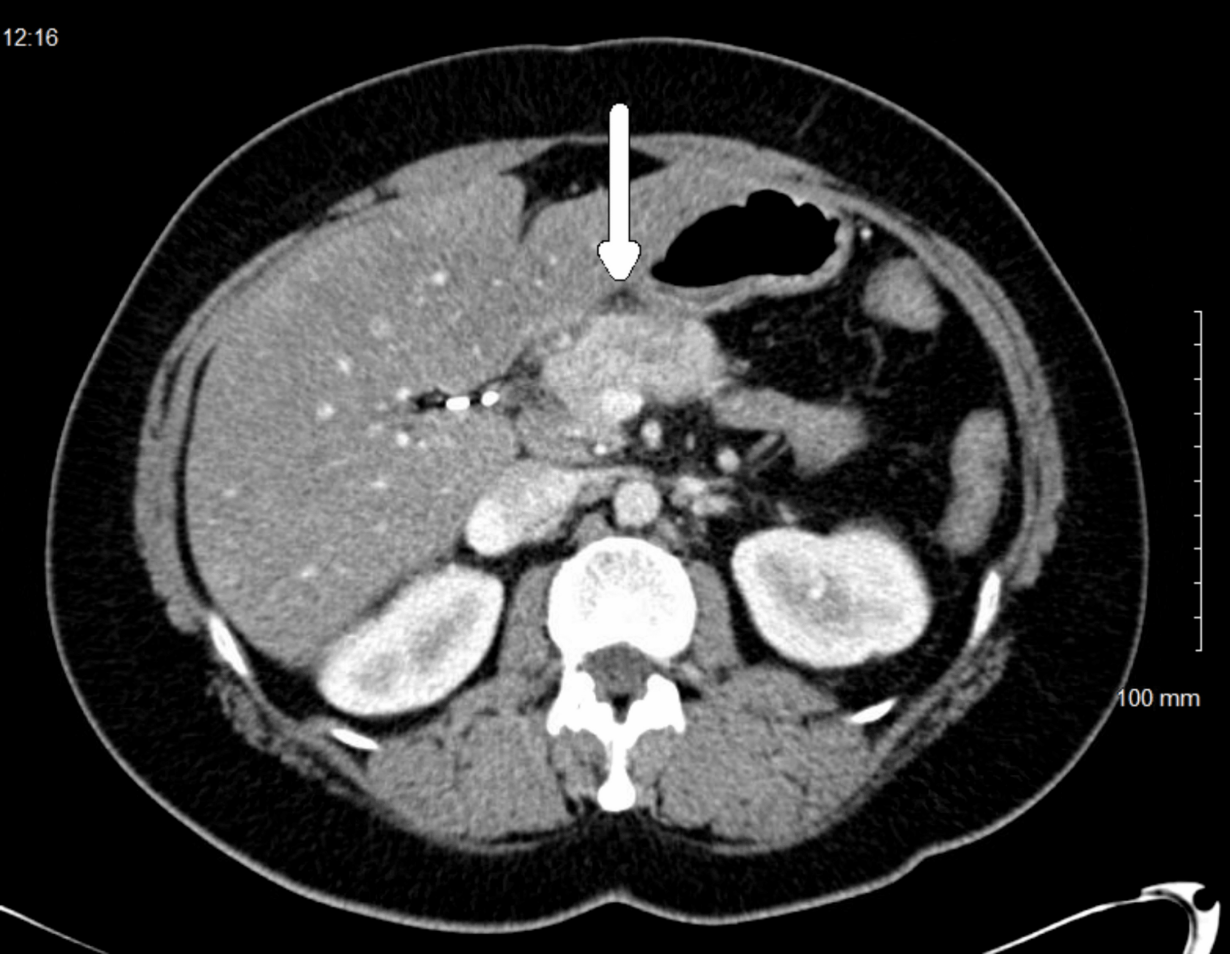
The CT showing a large enhancing mass in the pancreas (white arrow) CT: computed tomography

**Figure 2:**
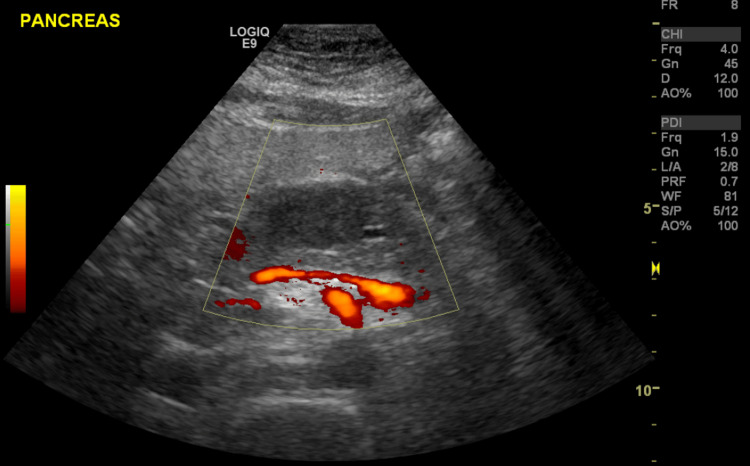
Ultrasound-guided biopsy of the pancreatic mass

In July 2014, a 54-year-old BRCA1-positive female presented to the emergency room for jaundice. A CT scan showed a 2.6 cm enhancing lesion in the pancreas and evidence of metastatic paraaortic and retroperitoneal lymph nodes (Figure 1). Ultrasound-guided pancreatic mass biopsy confirmed the diagnosis of poorly differentiated adenocarcinoma (Figure 2).

This case was discussed during a Multidisciplinary Cancer Conference (MCC) with the Princess Margaret Cancer Centre in Toronto, Canada, and was found to be unresectable due to involvement of retroperitoneal lymph nodes. The decision was made to treat with concurrent chemoradiation of 45 Gy in 25 fractions over five weeks with 5-FU as a radiation sensitizer in the first and fifth weeks.

**Figure 3:**
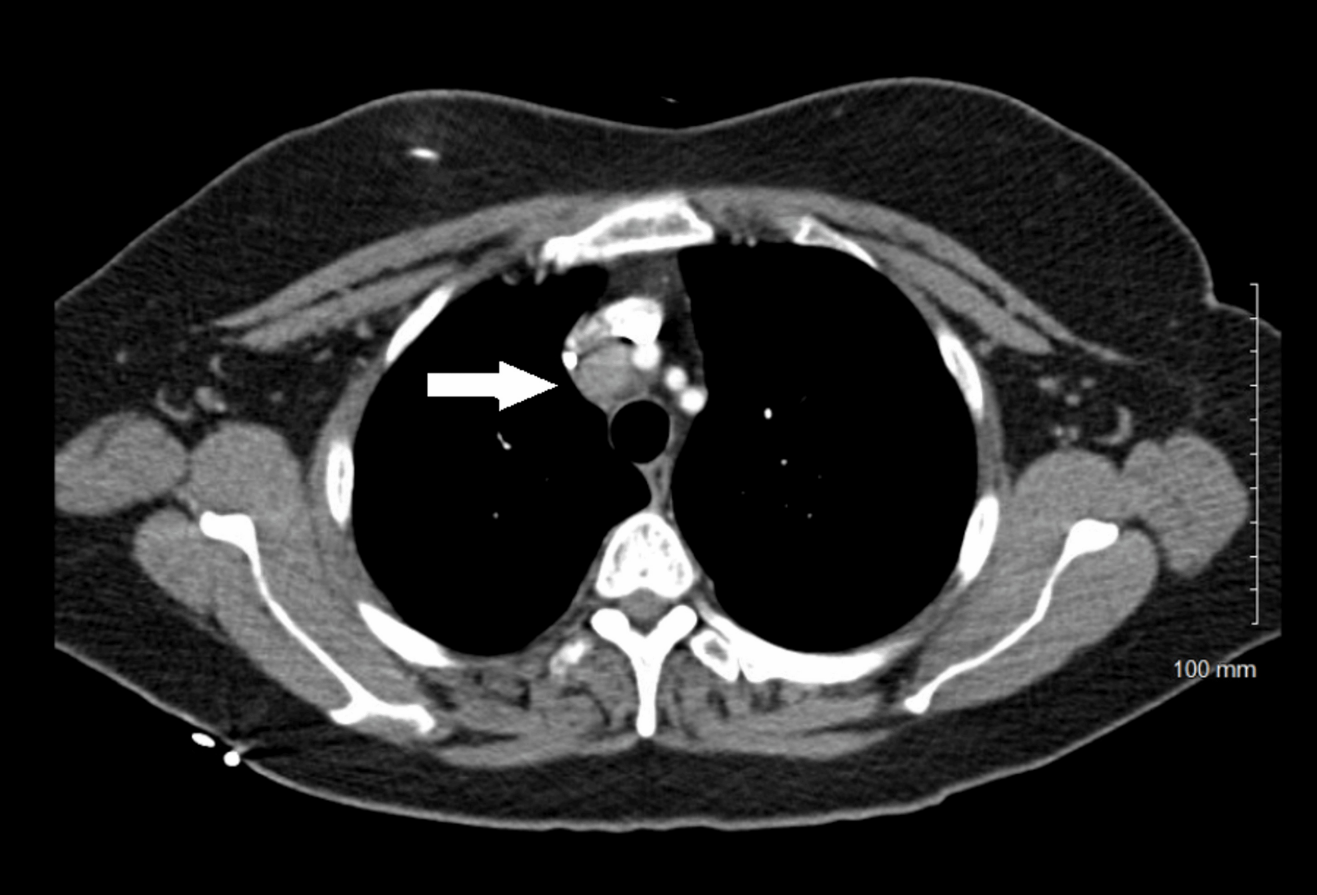
The CT showing metastases in station 2R of the mediastinum (white arrow) CT: computed tomography

**Figure 4:**
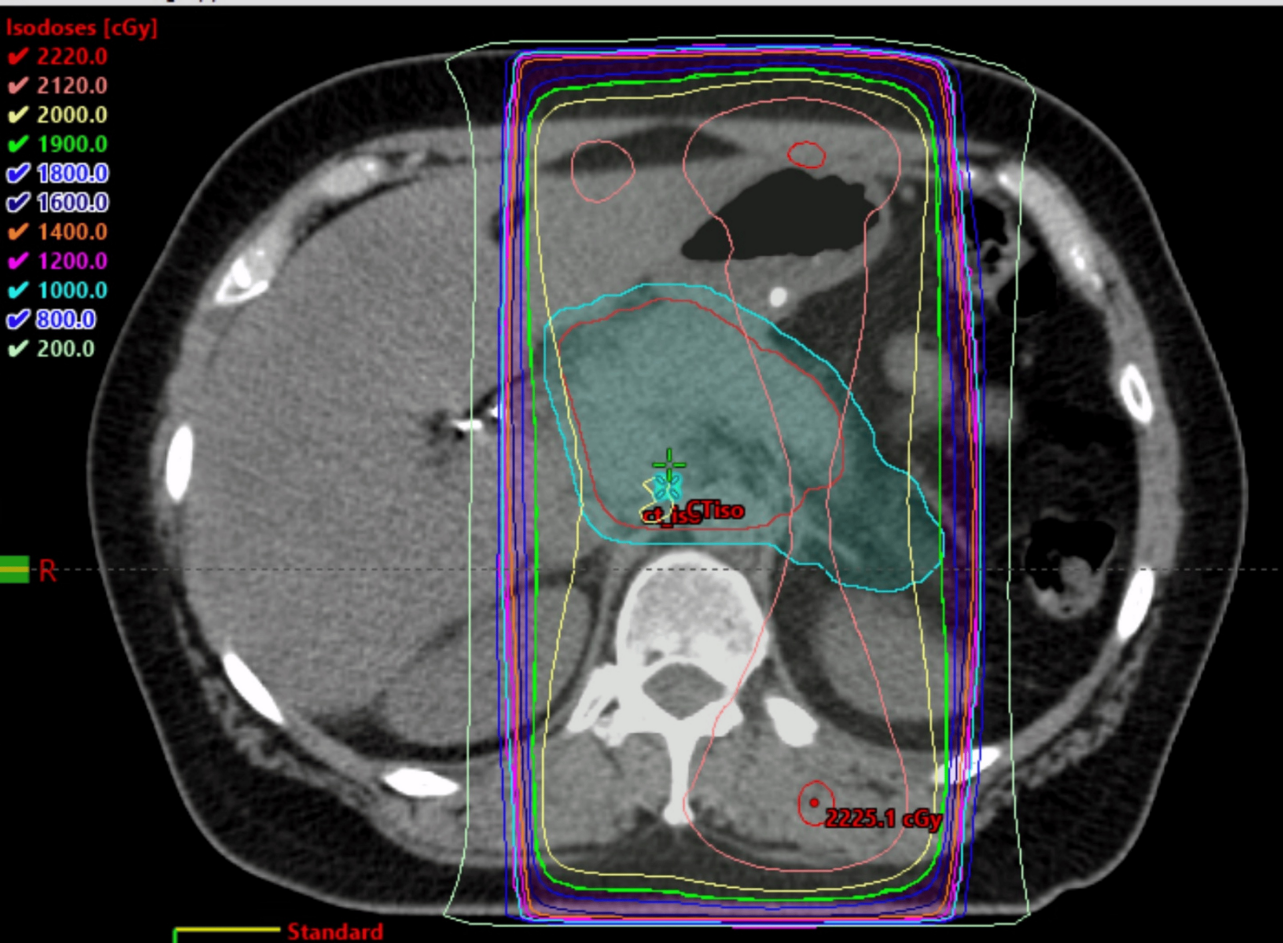
POP plan showing 100% dose of 20 Gy covering the primary cancer in pancreas POP: parallel opposed portals

A staging CT scan of the chest performed in September 2014, unfortunately, showed metastatic lymphadenopathy in station 2R of the mediastinum (1.4 cm in short axis) along with other smaller lymph nodes in the lower neck. The largest lymph node in the right hilar and paratracheal region was believed to be a metastasis from her known T3N1 pancreatic cancer, which put the patient in stage IV (Figure 3). She was informed that this was no longer curable. The treatment plan was changed to palliative radiation alone, 20 Gy in five fractions without chemotherapy, which was completed in September 2014 (Figure 4).

The patient then began palliative chemotherapy with a FOLFIRINOX regimen (folinic acid, fluorouracil, irinotecan, and oxaliplatin) from October 2014 to April 2015. Before her 11^th^ treatment, the patient faced some difficulties, such as diarrhea, gradual worsening of numbness in her fingers, and low neutrophils. For this reason, the chemotherapy dose was slightly reduced, and her 12^th^ treatment was postponed, allowing for blood cell count recovery. 

**Figure 5:**
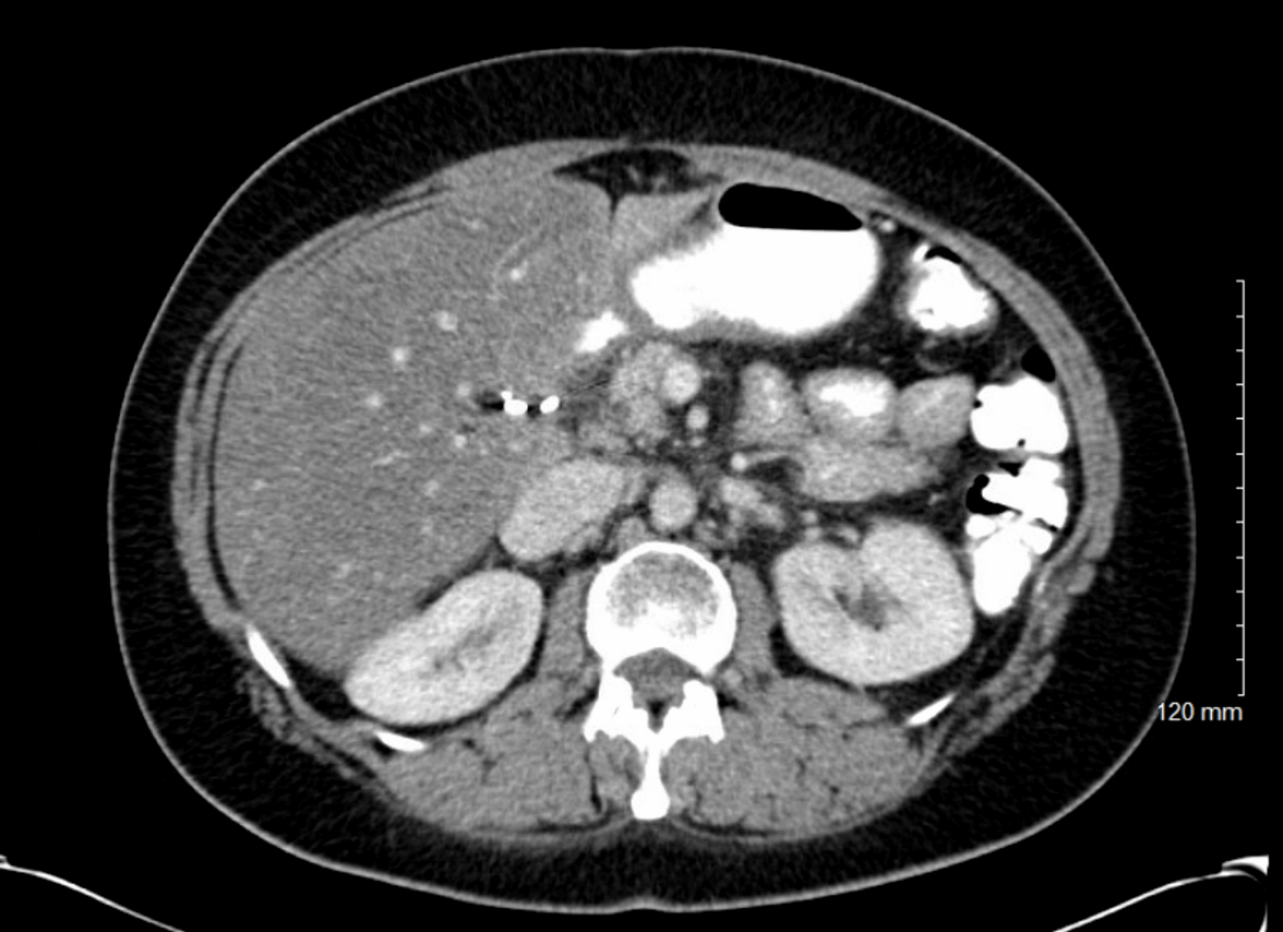
The CT showed complete response in the pancreas CT: computed tomography

CT imaging done in July 2015 showed a good response to treatment (Figure 5). The lesion in the body of the pancreas could no longer be seen. All mediastinal and retroperitoneal lymph nodes had responded to treatment, except for one small sub-centimeter lymph node near the porta hepatis. Her CA 19-9 level was still elevated at 87 kU/L; however, it had significantly decreased from previous values of 181 kU/L at the start of chemotherapy. No further treatments were deemed necessary at that time. She was simply followed closely.

**Figure 6:**
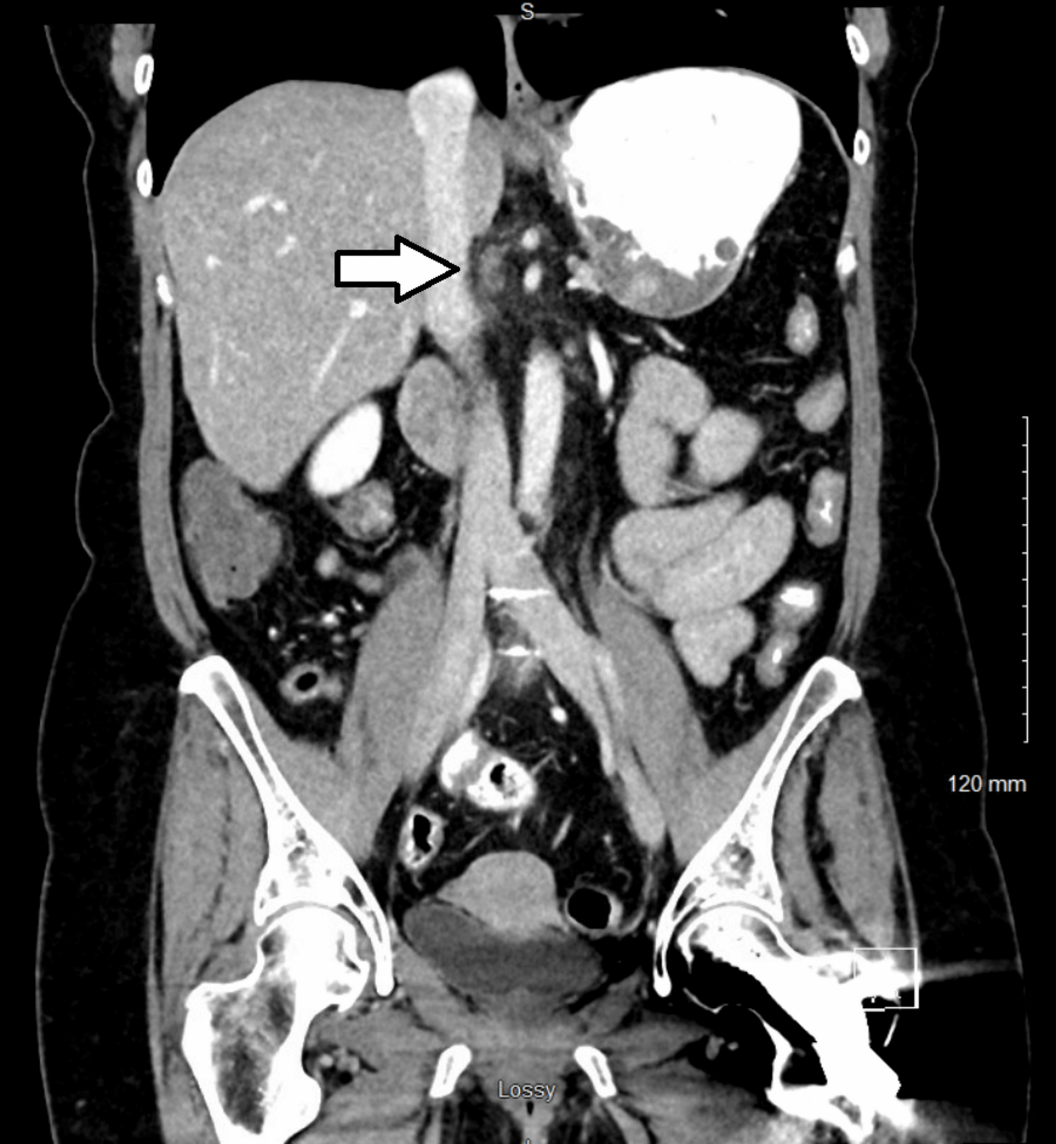
The CT showing one single growing portocaval lymph node (white arrow) CT: computed tomography

In 2021, almost seven years after receiving palliative radiation and chemotherapy, which achieved a near CR for her stage IV pancreatic adenocarcinoma, the patient presented with signs suggestive of disease progression. Follow-up CT imaging did not show any recurrent primary cancer or distant metastases, except for one single growing portocaval lymph node that measured 1.3 cm in the short axis (Figure 6). Her CA 19-9 levels had also begun to rise. This was discussed at the MCC, which concluded that the enlarged node was highly suspicious for oligometastasis but could not be safely biopsied. The options of positron emission tomography (PET) scan, watchful waiting, and palliative radiation treatment were discussed. 

**Figure 7:**
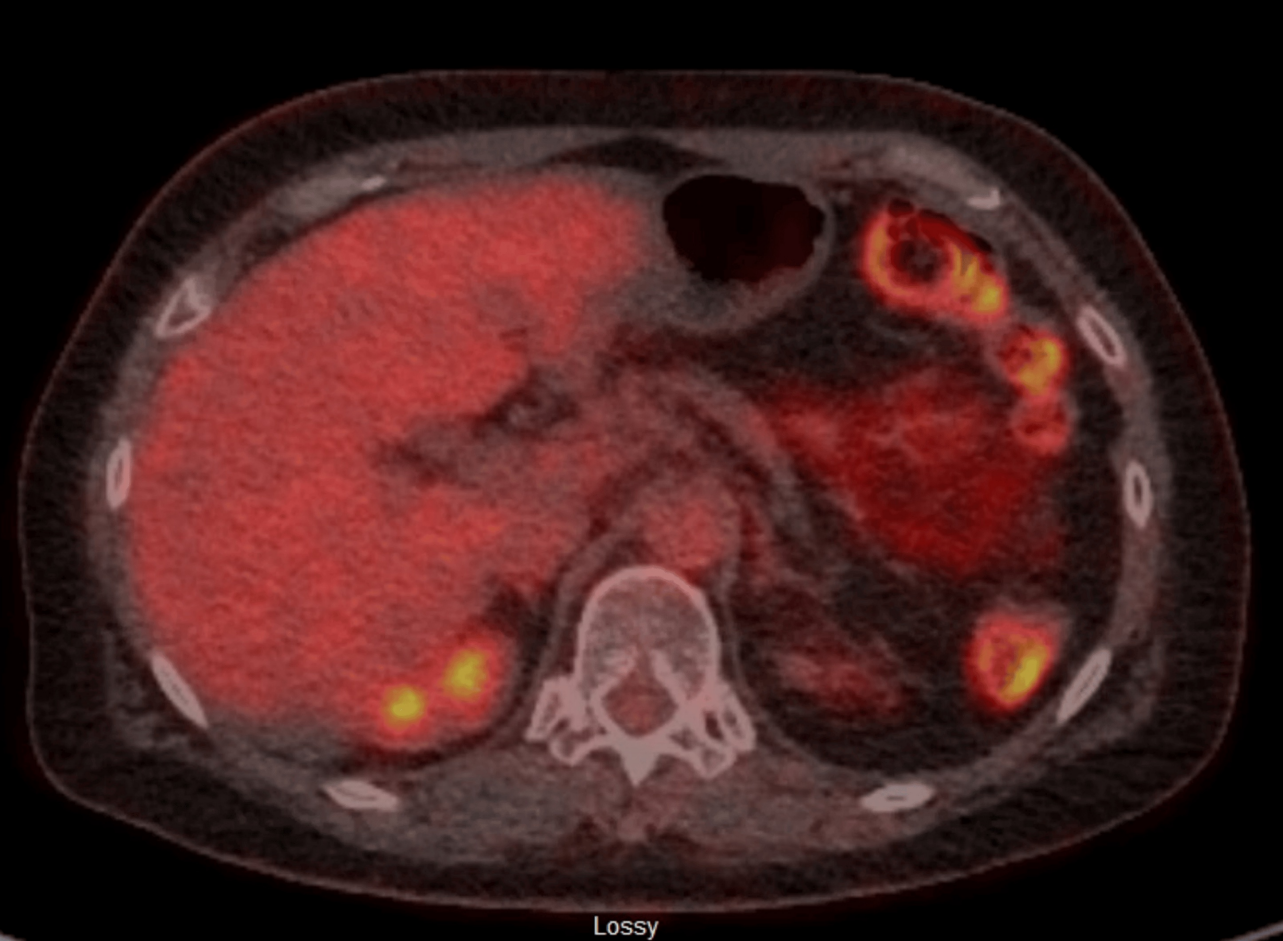
Post-treatment PET scan was negative for recurrence PET: positron emission tomography

A PET scan was completed in December 2021, which did not show any evidence of hypermetabolic primary cancer, lymph node, or distant metastases; this included the enlarging lymph node in the abdomen that measured 1.3 cm in the short axis (Figure 7). This case was again brought to MCC. Although possible radiation or stereotactic body radiation therapy (SBRT) to the single enlarged abdominal lymph node was discussed at the previous MCC, the experts at this MCC offered a different opinion. Since this single lymph node had been slowly growing over many years, the option of watchful waiting was raised again. Radiation was still an option, with the rationale to delay chemotherapy and the poorer quality of life that would result from systemic treatment. Repeating the tumor marker CA 19-9 and CT scan sooner, in three months, to re-evaluate was another option.

The three options were presented to the patient. Given that her response to treatment until now had been positive, she expressed extreme interest in trying radiation at this time to control the oligoprogression of the lymph node, lower the CA 19-9 levels if possible, and possibly further delay chemotherapy. 

**Figure 8:**
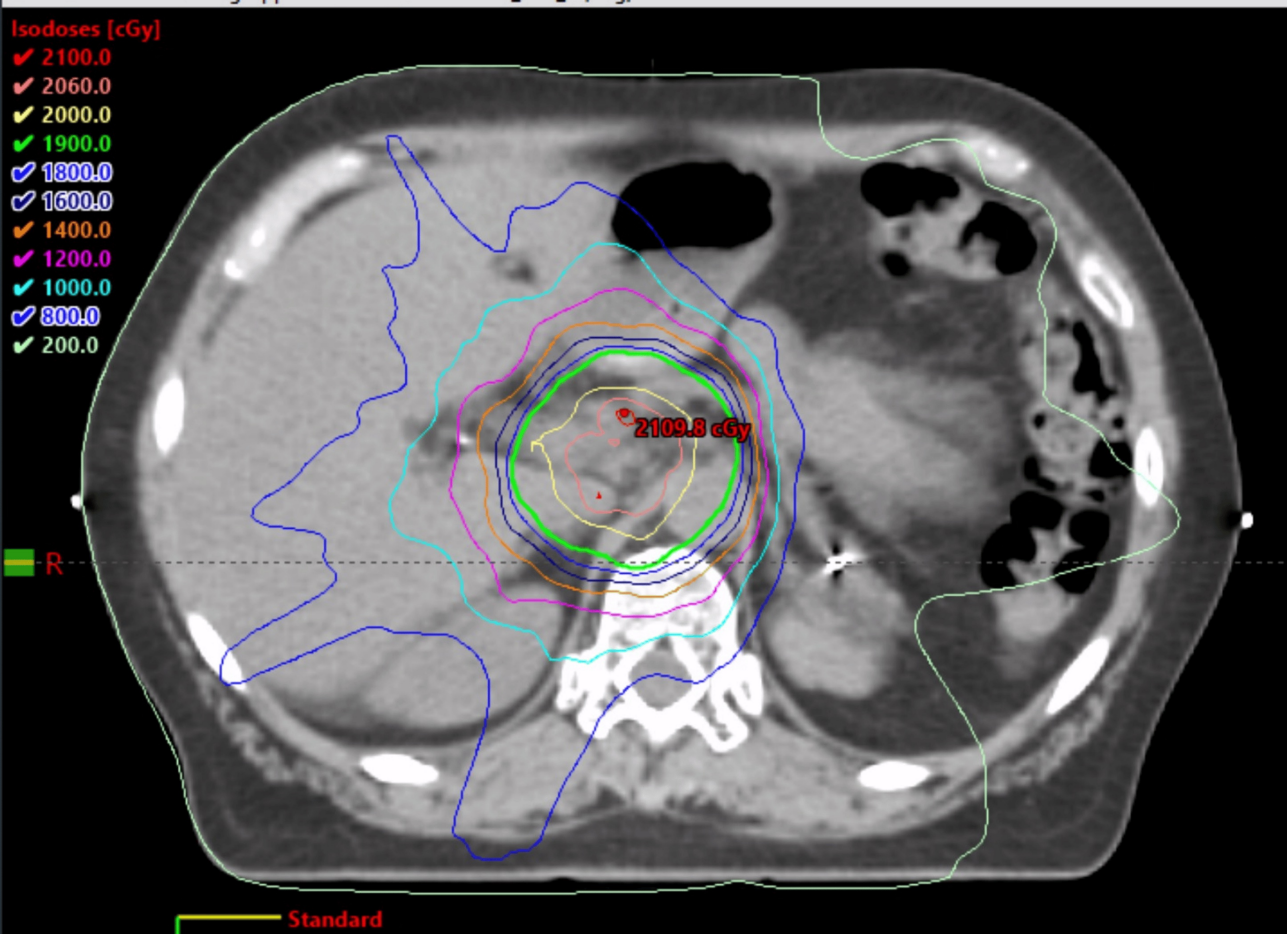
VMAT plan showing 100% dose of 20 Gy covering the oligo metastatic LN VMAT: volumetric modulated arc therapy; LN: lymph node

The patient received another palliative dose of radiation, 20 Gy in five fractions, to the abdominal lymph node in December 2021. We used volumetric modulated arc therapy (VMAT) to reduce the toxicities to the surrounding organs at risk (Figure 8). She did not have any side effects. She agreed to have a CT scan of the chest, abdomen, and pelvis, along with her CA 19-9 levels re-measured every three months.

**Figure 9:**
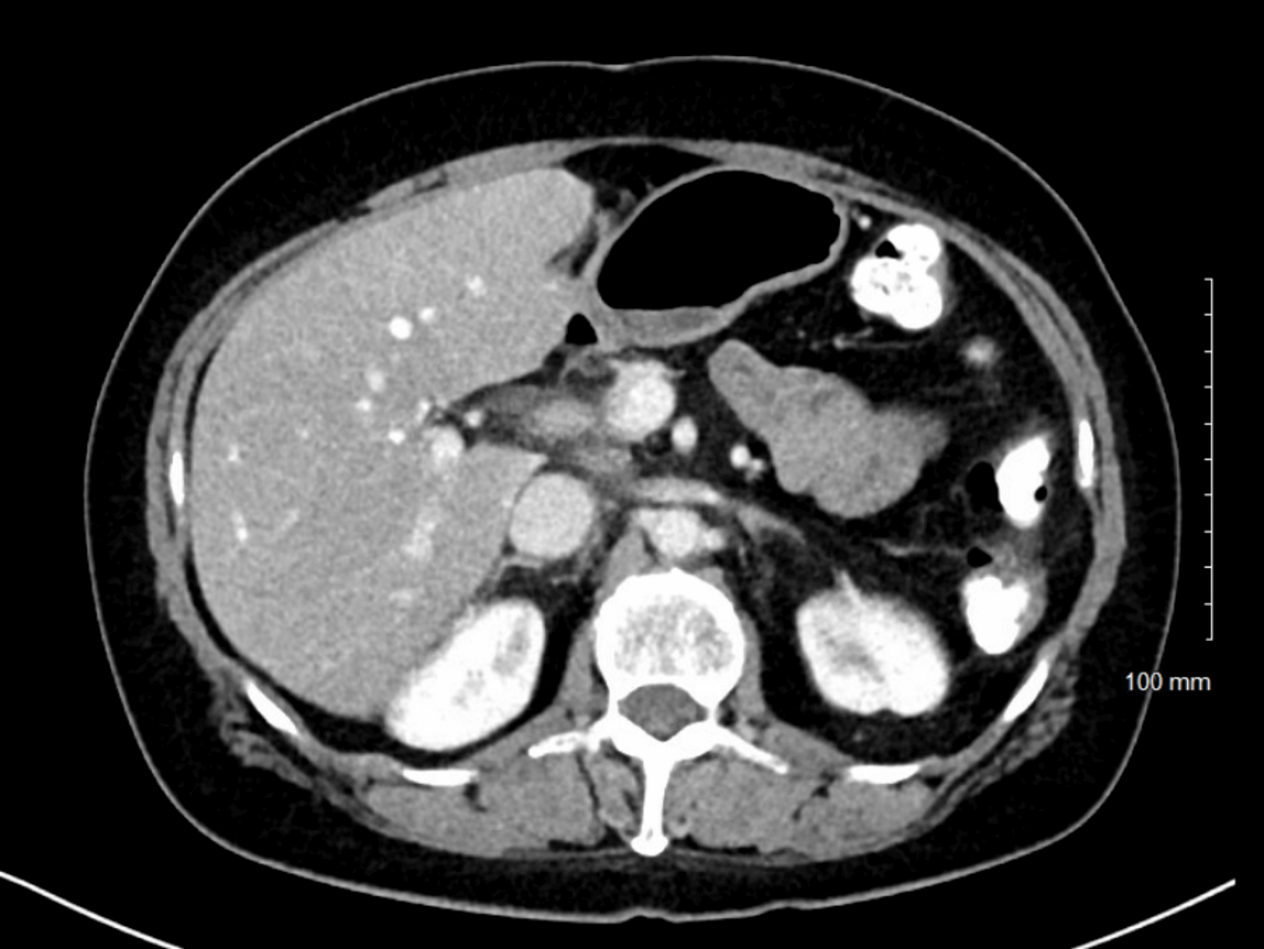
Post-VMAT CT reported as normal VMAT: volumetric modulated arc therapy; CT: computed tomography

Her first post-treatment CT scan was reported as normal (Figure 9). Her CA 19-9 did not significantly change; it was 89 kU/L before radiation and 88 kU/L after radiation. Most recently, a CT scan performed in November 2024 showed no local recurrence or metastasis. The stable small aortocaval lymph node was believed to be scar tissue or soft tissue fibrosis post radiation treatment from three years ago. 

**Figure 10:**
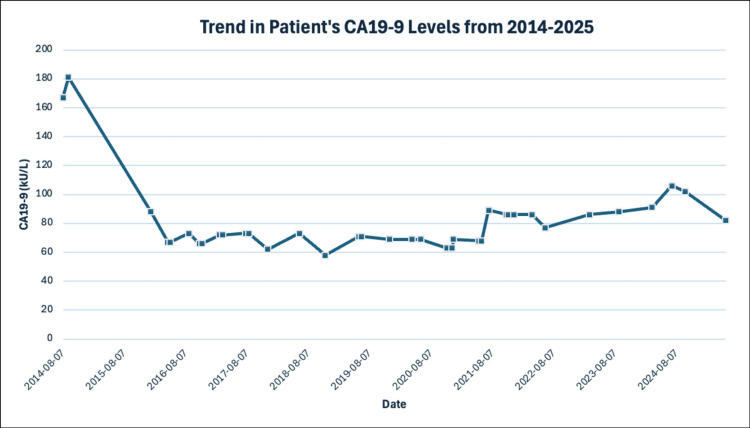
Trends in CA 19-9 values from 2014 to 2025 CA 19-9: serum carbohydrate antigen 19-9

On the last follow-up in November 2025, the patient was still alive without any symptoms or signs to suggest recurrent pancreatic cancer. There was no sign of any new cancer on her CT imaging and no elevations in CA 19-9 (stable at 82 kU/L in May 2025 and 75 kU/L in November 2025), which led the team to believe that this patient had likely been cured (Figure 10). Further radiation treatment was not recommended at this time, and the patient was not interested in chemotherapy due to severe toxicities endured in the past, nor was it currently warranted. The patient was happy with the results of the radiation and agreed to publish this case report to benefit future patients with pancreatic cancer.

## Discussion

To our knowledge, this is the first report of stage IV pancreatic adenocarcinoma being cured using only palliative dose radiation and FOLFIRINOX in a BRCA1-positive patient, more than 11 years after initial diagnosis. 

Oh et al. discussed the clinical progression of pancreatic adenocarcinoma survivors who did not undergo curative resection [6]. They found that only 2% (11/555) of non-resected pancreatic adenocarcinoma patients who received treatment lived more than five years. Specifically, one of their cases was BRCA2-positive and had a significant family history of cancer, but was still living 5.5 years post-initial diagnosis. All these patients had received five months of maintenance therapy on gemcitabine and docetaxel following their primary Virginia Mason Protocol chemoradiation regimen (interferon-alpha-2b). The longest OS reported at the time was 8.6 years [6].

However, recommendations for pancreatic cancer treatment have changed since the publication of their case series. The American Society of Clinical Oncology (ASCO) recommends FOLFIRINOX as first-line treatment for patients who have an Eastern Cooperative Oncology Group (ECOG) performance status (PS) of 0 to 1, conducive comorbidity profiles, and supports in place to manage various socioeconomic barriers [7]. Additionally, olaparib, a poly (ADP-ribose) polymerase (PARP) inhibitor, may be offered to BRCA1/BRCA2-positive patients who have had no progression of disease for a minimum of 16 weeks after platinum-based chemotherapy [7]. Our patient received only low-dose radiation and FOLFIRINOX after consideration of published literature and expert opinions presented at MCC rounds. Olaparib was not offered, as she had CR.

BRCA

Pancreatic cancer patients with BRCA1/BRCA2 mutations are uniquely positioned to benefit from platinum-based chemotherapies (PtCh) like FOLFIRINOX due to their tumors' defective homologous recombination repair (HRR) mechanisms, which leave them vulnerable to DNA-damaging agents [8]. These therapies exploit the inability of BRCA-mutated tumors to repair double-strand DNA breaks, leading to improved survival outcomes compared to patients without a BRCA mutation [9,10].

For instance, a pivotal retrospective study by Golan et al. revealed that among BRCA-mutated pancreatic ductal adenocarcinoma (PDAC) patients with stage III/IV disease, those treated with PtCh demonstrated a median overall survival (mOS) of 22 months, compared to just nine months for patients who did not receive platinum therapy (p = 0.039) [11]. This effect is consistent across platinum regimens, as demonstrated by Wattenberg et al., where BRCA-mutated patients receiving platinum therapy, including FOLFIRINOX, achieved an objective response rate (ORR) of 58%, compared to only 21% in matched controls (p = 0.0022) [12]. Moreover, findings from Memorial Sloan Kettering (MSK) showed a median progression-free survival (mPFS) of 12.6 months for platinum-treated patients versus just 4.4 months for non-platinum regimens (hazard ratio (HR) 0.44, p < 0.01) [13].

The importance of tailoring treatments for this molecular subgroup is further underscored by studies indicating no significant survival differences between BRCA1 and BRCA2 mutations, demonstrating that platinum therapies are broadly effective across both mutation types [10]. These results highlight the critical role of therapies like FOLFIRINOX in extending survival and improving response rates for BRCA-mutated PDAC patients, emphasizing the value of integrating genetic testing to identify these individuals early and guide optimal treatment.

CA 19-9

In stage IV pancreatic cancer, particularly among non-surgical candidates, CA 19-9 levels serve as a critical biomarker for prognosis and monitoring treatment response. Elevated baseline CA 19-9 levels are strongly correlated with worse survival outcomes, with studies consistently showing that patients with levels exceeding 37-200 kU/L have shorter median survival [14]. For example, a study demonstrated median survivals of 35 months for patients with CA 19-9 levels below 37 kU/L compared to just 16 months for levels above 200 kU/L [4]. Similarly, patients with CA 19-9 levels <37 kU/L showed a 60% 5-year disease-specific survival (DSS), while those with levels >37 kU/L had only a 4% 5-year DSS [4].

Dynamic changes in CA 19-9 levels during therapy are also predictive of outcomes. A ≥50% reduction in CA 19-9 values following neoadjuvant therapy was associated with significantly longer survival (42.3 months vs. 24.3 months) and improved recurrence-free survival (27.3 months vs. 14.1 months) [14]. Another study reported an 82% median reduction in CA 19-9 during chemotherapy, with patients achieving normalization (<50 kU/L) experiencing notably better survival compared to those without such reductions [15].

Persistently high or increasing CA 19-9 levels after treatment often indicate residual or metastatic disease, as suggested by their association with micro-metastases or perineural invasion [14].

Moreover, CA 19-9 trends may predict recurrence two to six months earlier than imaging, emphasizing its utility in guiding early therapeutic interventions [14]. While the sensitivity to benign conditions like pancreatitis and cholestasis can complicate interpretation, its consistent association with tumor dynamics across various thresholds reinforces its value [16]. However, the lack of standardized cut-off values and the need for supporting radiologic evidence currently limit its application in defining treatment pathways, underscoring the need for more comprehensive studies to solidify its clinical utility [14].

MCC

Long-term survivors of stage IV cancer patients have been occasionally reported in the past [17-20]. It is difficult to practice evidence-based medicine when there is very little level one evidence in the literature. MCC is very important in decision-making for these patients. All possible treatment options and potential outcomes should be discussed and provided to the patients for a better quality of life and sometimes even prolonged life expectancy.

## Conclusions

To our knowledge, the patient in this case is the longest survivor of stage IV BRCA1-positive pancreatic cancer ever reported, who has only received low-dose palliative radiation and chemotherapy. It demonstrated that the stability of CA 19-9 over time is more useful than the actual value in predicting recurrence and survival. Close follow-up with both CA 19-9 and CT imaging is important for this patient and saved her from unnecessary chemotherapy that carries significant toxicities. MCC helped us in decision-making to offer palliative treatment options to this terminal-stage pancreatic cancer patient, and we achieved surprisingly long survival.
